# The Disease-Modifying Effects of Hyaluronan in the Osteoarthritic Disease State

**DOI:** 10.1177/1179544117723611

**Published:** 2017-08-11

**Authors:** Mathew A Nicholls, Anke Fierlinger, Faizan Niazi, Mohit Bhandari

**Affiliations:** 1 Orthopedics and Sports Medicine, Virginia Mason, Seattle, WA, USA; 2 Ferring Pharmaceuticals Inc., Parsippany, NJ, USA; 3 Division of Orthopaedics, Department of Surgery, McMaster University, Hamilton, ON, Canada

**Keywords:** Knee, osteoarthritis, hyaluronic acid, mechanism of action

## Abstract

Hyaluronic acid (HA) has been a treatment modality for patients with knee osteoarthritis (OA) for many years now. Since HA was first introduced for the treatment of painful knee OA, much has been elucidated regarding both the etiology of this disease and the mechanisms by which HA may mitigate joint pain and tissue destruction. The objectives of this article are to (1) describe the etiology and pathophysiology of OA including both what is known about the genetics and biochemistry, (2) describe the role of HA on disease progression, (3) detail the antinociceptive and anti-inflammatory actions of HA in OA, and (4) present evidence of disease-modifying effects of HA in the preservation and restoration of the extracellular matrix. These data support that HA is not only just a simple device used for viscosupplementation but also a biologically active molecule that can affect the physiology of articular cartilage.

## Introduction

Over the past several decades, osteoarthritis (OA) of the knee and hip has emerged as the 11th leading cause of global disability.^1^ Among these 2 conditions, the prevalence of knee OA exceeds that of hip OA by several fold and it is estimated that nearly 1 in every 2 people will develop symptomatic knee joint OA by the age of 85 years.^1–3^ In the United States alone, more than 9 million adults have symptomatic OA of the knee.^4^ In particular, it is the senior population that is disproportionately affected, with 37.4% of adults more than the age of 60 years displaying radiographic evidence of this condition. Approximately, a third of these individuals (12.1%) suffer significantly from symptoms of pain and disability.^5^

Despite the high prevalence of knee OA in those more than 60 years in the United States, less than 2% of this population has a knee joint replacement, and the mean duration of time from disease onset to arthroplasty is 19 years.^5,6^ Such statistics underscore the continued importance of exploring and optimizing nonoperative treatments, such as hyaluronan (HA), which may have the potential to influence the biology of OA and ultimately improve the quality of life for millions of people having OA of the knee.

Hyaluronan has been used in thousands of patients for the treatment of painful OA of the knee, and its efficacy for this indication is supported by results from multiple clinical trials.^7–11^ Despite being classified in the United States, Europe, and other countries as a medical device to treat knee OA via intra-articular injection, it has been recognized that HA preparations may provide clinical benefit beyond boundary lubrication and shock absorption, via biochemical and genetic modifications that can attenuate nociceptive responses and blunt inflammation associated with OA.^12^ Since HA was first introduced for the treatment of painful knee OA, much has been elucidated regarding both the etiology of this disease and the mechanisms by which HA may mitigate joint pain and tissue destruction.

The objectives of this article are to (1) describe the etiology and pathophysiology of OA, (2) describe the role of HA on disease progression, (3) detail the antinociceptive and anti-inflammatory actions of HA in OA, and (4) present evidence of disease-modifying effects of HA in the preservation and restoration of the extracellular matrix (ECM).

## Etiology and Pathophysiology of OA

### Genetics and epigenetics

The etiology of OA is complex and involves both hereditary factors and alteration of gene expression within chondrocytes due to environmental and mechanical factors. Approximately 50% of the risk of developing OA is heritable, but only a few loci have been significantly associated with OA in genome-wide association studies.^13^ This may be due to epigenetic modifications, such as DNA methylation, histone modifications, and noncoding RNA, that can remodel chromatin and alter gene expression, thereby contributing to the development of OA.^13^ Environmental factors and mechanical stress have been shown to induce epigenetic changes within different cell types leading to pathology.^14^ In the field of vascular biology, cells that are constantly exposed to fluid shear stress and cyclic stretch from blood pressure undergo heritable genetic modifications that can influence physiology and pathophysiology.^14^ Similarly, the joint environment is subject to constant pressure and sheer stress, which may lead to heritable changes in chondrocytes and synoviocytes via mechanotransduction pathways. The link between epigenetic changes and OA continues to be investigated and connections continue to be further elucidated. For example, the OA disease state has been associated with the methylation status of promoter regions responsible for expression of cartilage-degrading proteins such as matrix metalloproteinases (MMPs) and inflammatory molecules.^15,16^ Given the central role of proteinases and inflammatory molecules in the progression of OA, exploring the mechanisms underlying their increased expression and their cartilage-destroying pathophysiology is paramount to understanding the therapeutic effects of interventions such as HA.

### Proteinases

#### Expression

Degradation of the ECM is a central feature of OA. In the OA joint environment, catabolic proteinases act on aggrecan and collagen, 2 essential components of the ECM.^17^ Polymorphisms in genes encoding MMP-1 and MMP-3 and A Disintegrin and Metalloprotease (ADAM)-12 have been shown to be associated with increased risk for the development of OA.^18,19^

Epigenetic changes in the regulation of genes encoding MMPs within synoviocytes and chondrocytes may also be involved in the development of OA. Significant promoter region demethylation of genes encoding MMP-3, MMP-9, and MMP-13, as well as A Disintegrin And Metalloproteinase with Thrombospondin Motifs (ADAMTS) 4, was found in chondrocytes extracted from patients with OA.^20^ These genes were also found to have methylated promoter regions in cartilage samples of patients without OA but who had suffered a femoral neck fracture.^20^ The demethylation positively correlated with the expression of these degrading enzymes.^20^ Reduced methylation of specific cytosine-phosphate-guanine (CpG) sites within the promoter region of MMP-13 and ADAMTS4 resulted in increased expression of these genes in the chondrocytes of patients with advanced OA and is consistent with the destructive effects of the enzymes that they encode.^21^ Epigenetic derepression associated with DNA methylation loss has also been demonstrated in chondrocytes affecting genes encoding MMP-3 and MMP-9.^22^

#### Pathophysiology of proteinases

Multiple studies have demonstrated increased levels of proteinases and aggrecanases in patients with OA.^17^ These enzymes cleave ECM proteins, including aggrecan and collagen, which may lead to chondral surface and structural damage.^17^ Hyaluronidase and reactive oxygen species (ROS) degrade HA into low-molecular-weight (LMW) fragments, and increased levels of LMW HA fragments are characteristic of later stage OA.^23–25^ Cross talk between subchondral bone osteoblasts and articular cartilage chondrocytes in OA alters the expression and regulation of a number of genes, including ADAMTS5, ADAMTS4, MMP-1, MMP-2, MMP-3, MMP-8, MMP-9, and MMP-13. These effects are mediated by the mitogen-activated protein kinase/extracellular signal–regulated kinase 1/2 signaling pathways.^26^ Indirect coculture of OA subchondral bone osteoblasts with normal articular cartilage chondrocytes resulted in a significant increase in the expression of ADAMTS5, ADAMTS4, MMP-2, MMP-3, and MMP-9, whereas coculture of OA articular cartilage chondrocytes led to increased MMP-1 and MMP-2 expression in normal subchondral bone osteoblasts.^26^

The increased expression of MMPs in chondrocytes from patients with OA, coupled with a growing understanding of the pathways involved in upregulation of these enzymes, has made them targets for OA therapies.^27^ Inhibition of MMP-13 may be of particular benefit due to its specific expression in the cartilage of patients with OA but not normal adult cartilage samples. Development of MMP-13–specific inhibitors has the potential to avoid the musculoskeletal side effects that have been associated with broad-spectrum MMP blockers.^28^

### Pro-inflammatory molecules

#### Expression

Inflammation plays a central role in the development and progression of OA, and there is a clear link between the progression of cartilage damage and the presence of a reactive or inflammatory synovium.^29^ Multiple inflammatory mediators are involved in OA, including interleukin (IL)-1β, IL-6, IL-15, IL-17, IL-18, IL-21, and tumor necrosis factor α (TNF-α).^29^ These cytokines act on multiple receptors, including CD44, HA-mediated motility receptor (RHAMM), and toll-like receptors (TLRs).^30,31^

The presence of inflammatory mediators is explained, at least in part, by increased genetic expression. Demethylation of specific CpG sites of the proximal IL-1β promoter in chondrocytes obtained from human cartilage has been correlated with increased expression levels for this gene.^16^ MicroRNAs also modify gene expression; it has been shown that miR-149 is downregulated in OA chondrocytes and that this decrease is associated with increased expression of the genes for pro-inflammatory cytokines, including IL-1β, TNF-α, and IL-6.^32^

#### Pathophysiology of inflammation

The cascade of inflammatory cytokines and other destructive molecules in OA has been well defined. Both IL-1β and TNF-α play key roles in cartilage destruction and could be considered as the initiators of the inflammatory cascade.^29^ IL-1β and TNF-α, produced by chondrocytes, mononuclear cells, osteoblasts, and synovial tissues, stimulate the production of many other inflammatory and catabolic molecules.^29^ IL-1β binds to receptors on chondrocytes and synovial cells and induces MMP synthesis. It also increases chondrocyte production of ADAMTS.^33^ In addition, IL-1β increases nitric oxide (NO) synthesis and decreases expression of the antioxidant enzymes that scavenge ROS, including superoxide dismutase, catalase, and glutathione peroxidase. The net effect of these changes may be an acceleration of the damaging effects of oxygen radicals on cartilage.^29,34^

Stimulation of chondrocytes by IL-1β also leads to the expression of TNF-α, which has many downstream pro-inflammatory effects.^29,33^ Tumor necrosis factor α suppresses the synthesis of proteoglycans and type II collagen in chondrocytes; stimulates the release of MMP-1, MMP-3, and MMP-13; and increases the production of IL-6, IL-8, monocyte chemotactic protein, and chemokine ligand 5.^29^

It has also been shown that IL-6, interferon-inducible protein 10, macrophage-derived chemokine, platelet-derived growth factor AA, and regulated on activation, normal T-cell expressed and secreted levels are higher in the synovial fluid from patients with OA as compared with that from normal controls (*P* < .001).^35^ Leptin, macrophage inflammatory protein 1β, and soluble CD40 levels are also elevated in synovial fluid from patients with OA vs that from normal subjects (*P* < .05).^35^

Interactions with these inflammatory mediators are the mechanism by which HA modifies the progression of OA. A detailed understanding of such interactions, as discussed in the following section, unequivocally highlights that the effects of HA extend beyond those of a “medical device” that simply lubricates and provides shock absorption.

## Role of HA on Disease Progression

Hyaluronan is a naturally occurring ECM molecule found in synovial fluid and is fairly ubiquitous throughout the body.^36^ It is a high-molecular-weight (HMW) glycosaminoglycan that is present at high levels in cartilage and synovial fluid and is believed to play an important role in joint lubrication.^37^ Hyaluronan also complexes with lubricin, a glycoprotein, to form a network that creates a boundary lubricant that decreases friction force and greatly reduces wear damage on rubbing/shearing surfaces.^37^ The lubrication provided by HA and lubricin is adaptive in that HA diffuses out of the cartilage during joint compression and becomes mechanically trapped at the joint interface by a constricted collagen pore network, thereby forming HA-lubricin complexes.^37^ Hyaluronan also endows synovial fluid with its viscoelastic properties.^38^

Altered characteristics of HA in the synovium can contribute to inflammation. Decreased HA synthesis, increased HA degradation, and elevated oxidative stress all lead to a decrease in both concentration and average molecular weight of the HA present in the synovium.^23,24^ Multiple studies have demonstrated that exposure of chondrocytes and fibroblasts to LMW HA fragments (<400 kDa) can cause an upregulation of pro-inflammatory cytokines.^39–41^ In addition, it has been shown that the levels of IL-18 and IL-33 are increased in mouse synovial fibroblasts after exposure to HA fragments,^40^ and that HA fragments enhance the inflammatory activity of macrophages.^42^ Table 1 summarizes the large number of pro-inflammatory molecules whose genes are induced by HA fragments and the cell types in which this occurs.^42^ In contrast, HMW HA appears to have the opposite effect on some of these systems, suppressing mediators such as TNF-α and IL-1β.^43–46^

**Table 1.** Selected genes that are induced by HA fragments and the cells in which this occurs.^42^

Category	Gene/protein	Cell type
Chemokines	CCL3	Macrophages
	CCL4	Macrophages
	CXCL2	Macrophages
	CCL5	Macrophages
	CCL2	Renal tubular epithelial cells
	CXCL10	Macrophage
	CXCL9	Macrophage
	CXCL1	Endothelial cells
	CCL5	Macrophages
	IL-8	Endothelial cells, epithelial cells
	CXCL1	Macrophages
Cytokines	IL-12	Macrophages, dendritic cells
	TNF-α	Dendritic cells
	IL-1β	Dendritic cells
Growth factors	TGF-β2	Monocytes
	IGF-I	Macrophages
Transcription factors	IκBα	Macrophages
	AP-1	Endothelial cells
	Rest	Monocytes
ECM	MMP-10	Endothelial cells
	MMP-13	Monocytes, dendritic cells
	PAI-1	Macrophages
	uPA	Macrophages
	MME	Macrophages
	MMP-9	Dendritic cells
	Collagen VIII	Endothelial cells
HSPG	Syndecan-4	Endothelial cells
Others	iNOS	Hepatocytes, endothelial, Kupffer, and stellate cells
	COX-2	Renal tubular epithelial cells
	MDR-1	Lymphocytes
	Trdn	Monocytes
	Frk	Monocytes

Abbreviations: AP-1, activator protein 1; CCL, chemokine ligand; COX-2, cyclooxygenase 2; CXC, chemokine receptor; ECM, extracellular matrix; Frk, fractalkine; HA, hyaluronan; HSPG, heparin sulfate proteoglycan; IGF-1, insulinlike growth factor 1; IκBα, nuclear factor of kappa light polypeptide gene enhancer in B-cells inhibitor α; IL, interleukin; iNOS, inducible nitric oxide synthase; MDR-1, multidrug resistance protein 1; MME, macrophage metalloelastase; MMP, matrix metalloproteinase; PAI-1, plasminogen activator inhibitor 1; TGF-β_2_, transforming growth factor β_2_; TNF-α, tumor necrosis factor α; Trdn, triadin; uPA, urokinase-type plasminogen activator.

Hyaluronan injection for treatment of OA of the knee is regulated by the US Food and Drug Administration (FDA) as a medical device; however, HMW HA, such as that used in viscosupplementation, also has multiple effects on molecular signaling pathways in several cell types found in synovial joints and contributes to the homeostasis of synovial joints (Table 2). The HA receptor activity may be responsible for the prolonged pain relief effect with intra-articular HA therapy, even though the residence time of the exogenous molecule within the joint is quite short.^54^

**Table 2.** Selected genes that are suppressed by HMW HA and the cells in which this occurs.

Category	Gene/protein	Cell type	References
Cytokines	IFN-γ	Chondrocytes	Campo et al, 2010^43^
	IL-1β	Chondrocytes	Chang et al, 2012^45^
		Macrophages	Baeva et al, 2014^46^
		Synoviocytes	Wang et al, 2006^44^
	IL-6	Chondrocytes	Campo et al, 2010^43^
		Macrophages	Yasuda et al, 2011^47^
		Synoviocytes	Wang et al, 2006^44^
	LIF	Synoviocytes	Wan et al, 2006^44^
	RANKL	Osteoblasts	Ariyoshi et al, 2014^48^
	TNF-α	Chondrocytes	Campo et al, 2010^43^
		Synoviocytes	Wang et al, 2006^44^
Chemokines	CCL5 (RANTES)	Chondrocytes	Tanaka et al, 2006^49^
	IL-8	Synoviocytes	Wang et al, 2006^44^
Transcription factors	Phospho-Akt/PKB	Macrophages	Yasuda et al, 2011^47^
	NF-κB	Chondrocytes	Chang et al, 2012^45^
		Macrophages	Yasuda et al, 2010^47^
	Phospho-JNK	Synoviocytes	Kataoka et al, 2013^50^
	Phospho-p38 MAPK	Chondrocytes	Yasuda, 2010^51^
		Synoviocytes	Kataoka et al, 2013^50^
	Phospho-ERK	Chondrocytes	Hashizume et al, 2010^52^
Proteases	ADAM17 (TACE)	Synoviocytes	Wang et al, 2006^44^
	ADAMTS4 (aggrecanase-1)	Synoviocytes	Wang et al, 2006; Kataoka et al, 2013^44,50^
	ADAMTS5 (aggrecanase-2)	Synoviocytes	Wang et al, 2006^44^
	MMP-1	Chondrocytes	Hashizume et al, 2010^52^
		Synoviocytes	Wang et al, 2006^44^
	MMP-2	Synoviocytes	Wang et al, 2006^44^
	MMP-3	Chondrocytes	Hashizume et al, 2010^52^
		Synoviocytes	Wang et al, 2006^44^
	MMP-9	Synoviocytes	Wang et al, 2006^44^
	MMP-13	Chondrocytes	Hashizume et al, 2010^52^
		Synoviocytes	Wang et al, 2006^44^
	TIMP-1	Synoviocytes	Wang et al, 2006^44^
	TIMP-2	Synoviocytes	Wang et al, 2006^44^
Others	COX-2	Macrophages	Yasuda et al, 2010^51^
	iNOS	Chondrocytes	Campo et al, 2010^43^
		Synoviocytes	Wang et al, 2006^44^
	TLR-4	Chondrocytes	Campo et al, 2010^53^

Abbreviations: ADAM, a disintegrin and metalloprotease; ADAMTS, a disintegrin and metalloproteinase with thrombospondin motifs; Akt, Ak strain transforming; CCL, chemokine ligand; COX-2, cyclooxygenase 2; ERK, extracellular signal–related kinase; HA, hyaluronan; HMW, high molecular weight; IFN-γ, interferon γ; IL, interleukin; iNOS, inducible nitric oxide synthase; JNK, c-jun N-terminal kinase; LIF, leukemia inhibitory factor; MAPK, mitogen-activated protein kinase; MMP, matrix metalloproteinase; NF-κB, nuclear factor κB; PKB, protein kinase B; RANKL, receptor activator of nuclear factor κB ligand; RANTES, regulated on activation; normal T cell expressed and secreted; TACE, tumor necrosis factor α–converting enzyme; TIMP, tissue inhibitor of metalloproteinases; TLR-4, toll-like receptor 4; TNF-α, tumor necrosis factor α.

## Antinociceptive and Anti-Inflammatory Actions of HA

### Antinociceptive effects

Hyaluronan products have certain rheologic properties that inhibit joint nociceptor discharges by acting as an elastoviscous filter. There is also a response to chemical sensitization of nociceptive terminals of inflamed joint tissues, possibly linked to HA concentration.^55^ Results from a study in cats with experimental arthritis indicated that intra-articular injection of HMW HA reduced the activity of pain-related primary afferents at baseline and during movement,^56^ suggesting that joint lubrication is not solely responsible for the antinociceptive effects of HA. Hyaluronan may coat pain receptors in synovial tissues and perhaps also trap molecules involved in pain signaling.^57^ Recently, single intra-articular injections of HA were shown to decrease pain by more than 50% compared with saline in a bradykinin/prostaglandin E_2_ (PGE_2_) model.^58^ In addition, a single injection of HA-attenuated pain responses for at least 56 days after administration.^58^ Injection of HA into the superior compartment of the temporomandibular joint in patients with unilateral internal derangement has been shown to significantly decrease pain (*P* < .01), as well as joint levels of leukotriene C_4_, 6-keto-prostaglandin F_1_α, prostaglandin F_2_α, and IL-1β (all *P*’s < .05).^59^ Experiments using human macrophages have indicated that HMW HA interferes with lipopolysaccharide (LPS)-induced increases in the production of PGE_2_ and cyclooxygenase 2 (COX-2). In these cells, pretreatment with HA suppressed induction of COX-2, leading to a decrease in PGE_2_ production. Use of an anti-CD44 antibody reversed the inhibitory effects of HA on the LPS-mediated increase in PGE_2_ production and COX-2 induction, indicating that the anti-inflammatory effects of HA were CD44 receptor mediated.^51^

Additional mechanisms that may contribute to the antinociceptive effects of HA include an inhibition of arachidonic acid release from fibroblasts and activation of opioid receptors.^60,61^ Exposure to HA has been shown to decrease secretion of arachidonic acid from fibroblasts taken from patients with knee OA and stimulated with bradykinin or induced by a calcium ionophore.^60^ More recent in vitro experiments demonstrated that HA stimulated κ opioid receptors expressed by Chinese hamster ovary cells and rat dorsal root ganglion neurons.^61^

Results from one study have indicated that unlike anti-inflammatory drugs, pain reduction resulting from HA administration was associated with cartilage preservation. Both HA and loxoprofen significantly decreased pain in a rabbit OA model (partial meniscectomy), as measured by hind paw weight distribution, and they also reduced PGE_2_ production. Hyaluronan treatment also significantly inhibited cartilage degeneration, whereas loxoprofen did not.^52^

### Anti-inflammatory effects

High-molecular-weight HA has the potential to inhibit the inflammatory events involved in OA by interfering with the actions of LMW HA fragments at CD44, RHAMM, and TLR-2 and TLR-4. Results from in vitro and in vivo studies indicate that administration of HMW HA has significant anti-inflammatory effects that are mediated, at least in part, by blockade of CD44. Administration of HMW HA leads to the downregulation of IL-8 and inducible NO synthase gene expression in cells that were not stimulated with IL-1. In cells that were stimulated with IL-1, TNF-α gene expression was also downregulated. Blocking CD44 with a specific antibody inhibited the effects of HMW HA on pro-inflammatory gene expression.^44,62,63^ It has also recently been shown that HMW HA suppresses IL-1β production in monocyte/macrophage cultures under various inflammatory conditions.^46^

Inhibition of IL-1β and TNF-α production by HA has important downstream effects on the expression of pro-inflammatory and catabolic molecules. IL-1β induces ADAMTS via p38 mitogen-activated protein kinase and c-jun NH2-terminal kinase phosphorylation in human fibroblast-like synoviocytes. The ADAMTS degrade aggrecan in cartilage; HMW HA also suppresses ADAMTS expression.^50^

IL-1β downregulates peroxisome proliferator–activated receptor γ (PPARγ) and increases expression of MMPs.^45^ Results from a study focused on inflammatory gene expression in IL-1β–stimulated human chondrosarcoma cells indicate that HMW HA increases the expression of PPARγ and decreases that for COX-2, MMP-1, and MMP-13. Additional anti-inflammatory effects of HA demonstrated in this study included suppression of mitogen-activated protein kinases and nuclear factor κB signaling.^45^

In a rabbit model that induced OA through sterile papain solution injection into the knee, an intra-articular injection of HA resulted in significant reductions in the expression of IL-1β and TNF-α and increased expression of TIMP-1 compared with intra-articular saline-treated controls.^64^ Histologic analysis and Mankin scores also indicated significantly less degeneration in the HA-treated vs control animals (*P* < .01). Treatment with HA also resulted in proliferation of chondrocytes in this model.^64^

Exogenous HA also decreases levels of inflammatory cytokines and MMPs in tissues taken from patients with OA and other conditions associated with joint damage. In one study, subacromial synovium fibroblasts were taken from patients with rotator cuff disease and stimulated with IL-1β. Addition of HA resulted in a dose-dependent decrease in the expression of IL-1β, TNF-α, and IL-6. These effects of HA were lost when CD44 was blocked with the anti-CD44 antibody, OS/37.^65^ Incubation with HA has also been shown to inhibit IL-1β–induced MMP activity in explants of synovial tissue from patients with OA. This effect was also potentially mediated by interaction of HA with CD44.^66^

Oxidative stress resulting from chronic overproduction of ROS plays an important role in OA, and this stress may be induced by abnormal cyclic loading of the joint.^67,68^ Free radicals can damage DNA, decrease cell viability, and contribute to disruption of the ECM. Reactive oxygen species reduce synthesis of proteoglycans and accelerate chondrocyte senescence and thus their ability to repair tissue.^68^

Results from several studies have demonstrated that HA reduces levels of ROS and also protects chondrocytes from the adverse consequences of exposure to these molecules. Several experiments have shown that HA can decrease ROS production resulting from mechanical stress. For example, mechanical compression of bovine cartilage increases ROS production, and this effect is attenuated by incubating the tissue with HA.^69^ In a rabbit model in which OA was induced by intra-articular injection of papain, treatment with HA significantly decreased the expression of NO vs control animals treated with saline (*P* < .05).^64^ It has also been shown that HA inhibits NO-induced apoptosis and dedifferentiation of chondrocytes in vitro.^70^

Increased expression of MMPs and aggrecanases contributes to the cartilage-destructive characteristic of OA,^71^ and intra-articular administration of HA has been shown to significantly inhibit these proteases. In human chondrocytes, stimulation with IL-6 and the IL-6 soluble receptor increases expression of MMP-1, MMP-3, and MMP-13. The effect is inhibited by HA via the CD44 receptor. In vitro incubation of human chondrocytes with an anti-CD44 antibody blocks this action, indicating that interaction of HA with CD44 is necessary for inhibition of MMP upregulation.^72^ In a rabbit model of OA induced by anterior cruciate ligament transection, intra-articular injection of HA decreased OA severity and suppressed expression of MMP-13 in subchondral bone. These effects also required interaction of HA with CD44.^73^ In a different rabbit OA model that induced disease by injection of papain, treatment with HA significantly increased synovial fluid levels of TIMP-1, an inhibitor of MMPs.^64^

Furthermore, HA has been shown to decrease MMP expression in patients with OA. In a trial of 51 patients with knee OA who received intra-articular injections of HA or chondroitin sulfate and were followed for 6 months, both treatments significantly decreased pain/inflammation scores (*P* < .01). However, only HA significantly reduced levels of MMP-9 (*P* < .01).^74^

## Disease-Modifying Effects of OA—Tissue Protection

### Exogenous HA—preservation and restoration of the ECM

During the progression of OA, cartilage ECM is remodeled by proteases expressed by chondrocytes in response to inflammation. Changes in the ECM alter the biomechanical environment of chondrocytes and result in disease progression.^75^ The ECM is integrally involved in the development and progression of OA, and its preservation and restoration have become the focal point of treatment.

Results from several studies have indicated that exogenous HA can increase the synthesis of ECM molecules.^74,76^ Exogenous HA stimulates synovial fibroblasts to produce new HA. When synovial fibroblasts from OA knees were cultured with HA formulations of various molecular weights, the amount of newly synthesized HA was dependent on both the concentration and molecular weight of the exogenous HA. Higher molecular weight agents stimulated more HA synthesis and very LMW HA suppressed HA synthesis when applied at high concentrations.^25^ Two additional studies have shown that intra-articular injection of HA in patients with OA increases endogenous HA production.^74,76^

In vitro experiments that treated bovine articular chondrocytes with HA induced a significant increase in sulfated glycosaminoglycan and hydroxyproline synthesis, which was coincident with increased matrix deposition of chondroitin-6-sulfate and collagen type II.^77^ Mechanical stress resulting in injury has been shown to result in loss of proteoglycans from cartilage and can play a role in the development and progression of OA,^78^ whereas administration of HA has been shown to increase proteoglycan synthesis in cartilage subjected to mechanical stress.^69^

Osteopontin is an extracellular scaffold protein that is upregulated in OA cartilage and inhibits IL-1β–induced NO and PGE_2_ production in human OA–affected cartilage in response to joint inflammation.^79^ It has been shown that exposure to HA significantly increases osteopontin expression in fibroblast-like synoviocytes from patients with OA of the knee, thus potentially amplifying its anti-inflammatory actions.^80^

### Exogenous HA—clinical evidence of tissue protection

Although there is a host of basic science and animal studies that demonstrate the ability of HA to significantly blunt rheologic, inflammatory, and physical changes brought about by OA, there has been less focus on support of this potential clinically in human studies. In 2007, a review of disease-modifying OA drugs dedicated only a single paragraph to HA, concluding that there was no evidence that HA provided a disease-modifying effect.^81^ This conclusion was based on the observation that intra-articular HA injection into the knee had no significant effect on radiographic progression vs intra-articular saline over 1 year of follow-up.^82^ However, a subgroup analysis of patients with less severe disease at baseline indicated significantly less joint space narrowing with HA vs saline (*P* = .02).^82^ Ultimately, however, there is a growing consensus that plain film radiographs may be insensitive to potential disease-modifying effects of OA treatments that are currently used as well as those in development.^83^

As imaging technology becomes more advanced, the macroscopic impact of HA supplementation in both experimental animals and clinical patients will become more evident. For example, in a study that employed an anterior cruciate ligament transection model of OA in rats, T2-weighted magnetic resonance (MR) imaging was used to evaluate the effects of intra-articular injection of HA, intra-articular saline, and sham injections. Study results indicated significant superiority of intra-articular HA over intra-articular saline for T2 MR values (*P* < .05). Study results also showed that the T2 values were significantly and positively correlated with Mankin scores.^84^ Results from a study of patients evaluated with T1ρ and T2 MR imaging just prior to total knee arthroplasty indicated T1ρ mapping was superior to T2 mapping for evaluation of denatured articular cartilage associated with OA of the knee.^85^ A clinical trial using T1ρ MR imaging to evaluate effects of intra-articular HA injection is currently under way. Further human studies are needed to demonstrate that these basic science principles can indeed translate into disease modification in human OA pathology.

The importance of evaluating outcomes relevant to the patient, such as pain and quality of life, alongside imaging, cannot be overstated, as approximately two-thirds of adult patients with radiographic evidence of knee OA are asymptomatic.^5^ Since 2007, studies have investigated the potentially beneficial effects of HA in relieving OA pain and reducing tissue destruction.^55^ A 2006 Cochrane Review of randomized trials concluded that viscosupplementation with HA (or hylan derivatives) was superior to placebo in improving pain and function at several weeks. Furthermore, viscosupplementation generally demonstrated benefit for a longer duration compared with intra-articular corticosteroid injections.^86^ More recent systematic reviews have also shown safety and efficacy of HA over nonsteroidal anti-inflammatory drugs and other nonoperative treatment modalities.^87,88^

## Conclusions

**Figure 1.**
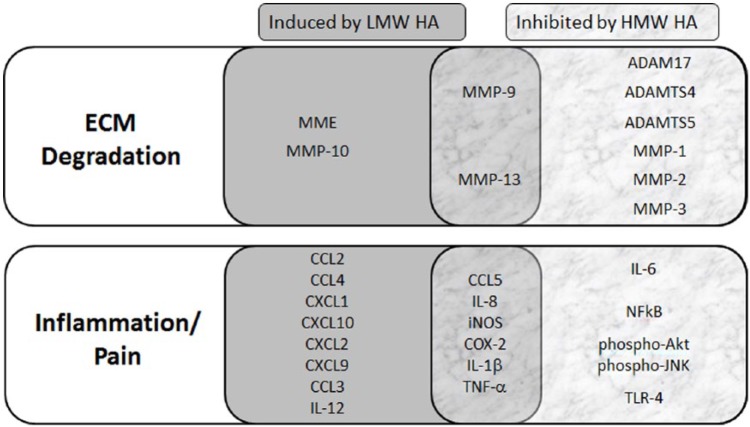
Selected genes that are involved in ECM degradation (top) and inflammation/pain (bottom) responses in the OA environment. Data on protein function were obtained by searching the Universal Protein Resource (UniProt; www.uniprot.org) and analyzing the gene ontology associated with each protein. Genes within the gray (left) box are induced by LMW HA fragments and those within the marble (right) box are inhibited by HMW HA. The overlapping region (center) show selected genes that have been shown to be both induced by LMW HA and inhibited by HMW HA. ADAM indicates a disintegrin and metalloprotease; ADAMTS, a disintegrin and metalloproteinase with thrombospondin motifs; Akt, Ak strain transforming; CCL, chemokine ligand; COX-2, cyclooxygenase 2; CXC, chemokine receptor; ECM, extracellular matrix; HA, hyaluronan; HMW, high molecular weight; IL, interleukin; iNOS, inducible nitric oxide synthase; JNK, c-jun N-terminal kinase; LMW, low molecular weight; MME, macrophage metalloelastase; MMP, matrix metalloproteinase; NF-κB, nuclear factor κB; OA, osteoarthritis; TLR-4, toll-like receptor 4; TNF-α, tumor necrosis factor α.

Hyaluronan products used for viscosupplementation are considered medical devices by the FDA. It has become increasingly apparent that HA influences a wide range of biologic processes via multiple molecular pathways (Figure 1). This review presents evidence for the broader role of HA in the treatment of OA beyond joint cushioning and lubrication. Exogenous HA can reduce pain transmission and blunt the inflammatory cascade via the CD44 receptor that is associated with OA, as well as stimulate synthesis and deposition of ECM molecules that are suppressed and degraded in an osteoarthritic joint. These data also show that the effect of HA is dependent on the size of the fragment. In particular, long-chain HMW HA exerts an anti-inflammatory effect and can stimulate the production of endogenous HA, whereas shorter HA fragments are pro-inflammatory and can inhibit HA production at high concentrations. Although there are many molecules that will reduce the pain and inflammation due to OA, HA has the potential to reduce pain as well as to protect and restore the chondral matrix.

Much of what is known regarding the biochemical actions of HA in the knee OA environment comes from experiments with animal models and human explants. Only a small number of clinical studies have evaluated the biochemical effects of HA in patients with OA. Large-scale clinical trials that evaluate biomarker changes in response to treatment, as well as noninvasive imaging studies, would be beneficial for further elucidating the mechanism(s) of action of HA in OA, demonstrating disease modification in vivo and providing insight into additional biological targets for treatment of this very common disease.
